# Chromosome-level genome assembly of *Euphorbia tirucalli* (Euphorbiaceae), a highly stress-tolerant oil plant

**DOI:** 10.1038/s41597-024-03503-w

**Published:** 2024-06-21

**Authors:** Zuoying Wei, Chao Feng, Jiayun Xu, Xizuo Shi, Ming Kang, Jing Wang

**Affiliations:** 1 State Key Laboratory of Plant Diversity and Specialty Crops, South China Botanical Garden, Chinese Academy of Sciences, Guangzhou, 510650 China; 2 University of Chinese Academy of Sciences, Beijing, China; 3 South China National Botanical Garden, Chinese Academy of Sciences (CAS), Guangzhou, China; 4 Key Laboratory of National Forestry and Grassland Administration on Plant Conservation and Utilization in Southern China, Guangzhou, 510650 China

**Keywords:** Genome evolution, Biogas

## Abstract

*Euphorbia*, one of the largest genera of flowering plants, is well-known for containing many biofuel crops. *Euphorbia tirucalli*, an evergreen succulent mainly native to the Africa continent but cultivated worldwide, is a promising petroleum plant with high tolerance to drought and salt stress. However, the exploration of such an important plant resource is severely hampered by the lack of a reference genome. Here, we present the chromosome-level genome assembly of *E. tirucalli* using PacBio HiFi sequencing and Hi-C technology. Its genome size was approximately 745.62 Mb, with a contig N50 of 74.16 Mb. A total of 743.63 Mb (99.73%) of the assembled sequences were anchored to 10 chromosomes with a complete BUSCO score of 97.80%. Genome annotation revealed 26,304 protein-coding genes, and 76.37% of the genome was identified as repeat elements. The high-quality genome provides valuable genetic resources that would be useful for unraveling the genetic mechanisms of biofuel synthesis and evolutionary adaptation of *E. tirucalli*.

## Background & Summary

**Fig. 1:**
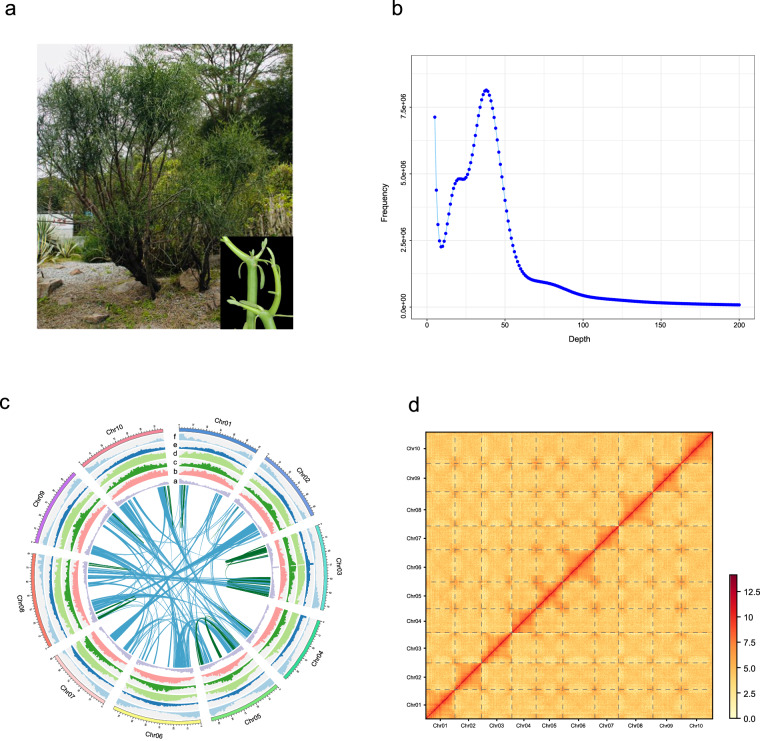
Overview of *Euphorbia tirucalli* genome assembly and features. (**a**) The picture of the sequenced *E. tirucalli* from South China National Botanical Garden (accession number: IBSC0312991). The inset map shows its succulent branched stems and small non-succulent leaves. (**b**) *K*-mer (17-mer) frequency distribution curve. (**c**) Distribution of genomic features of *E. tirucalli*. Tracks ‘a–f’ represent tandem repeat density, LTR *Gypsy* density, LTR *Copia* density, TE density, GC content, and gene density, respectively. (**d**) Hi-C interaction heat map for *E. tirucalli*.

*Euphorbia*, belonging to the family Euphorbiaceae, comprises about 2000 species and is one of the largest flowering plant genera in the world^1^. Many fuel plants have been reported in this genus, providing biomass for the production of biocrude, bioethanol and other bioenergy resources^2–5^. *Euphorbia tirucalli* (2n = 2x = 20)^6^, commonly referred to as milk bush, pencil cactus, pencil tree, or naked lady, is an evergreen shrub or small tree with typically succulent branched stems and small non-succulent leaves (Fig. 1a). It is naturally distributed in Indochina, South Africa, East Africa and Madagascar, and has been extensively cultivated as horticultural plant in other tropical or subtropical areas^7^. As one of the representative oil plants, *E. tirucalli* has long been considered a promising substitute for traditional energy sources. It exudes a milky latex from the wounded shoots or leaf stems^3,8^. Recent studies demonstrated that the compounds in the latex exhibit high petrochemical properties^5,9,10^. In addition, this species has important medicinal value, with its latex being traditionally used for the treatment of cancer, asthma, arthritis, rheumatism and so on^11–14^.

*Euphorbia tirucalli* has high salinity and drought tolerance, which enables it to grow under a wide range of adverse conditions without occupying any arable land. Different genotypes exhibit distinct evolutionary adaptation to environmental stress^15^. In particular, the special photosynthetic system in *E. tirucalli*, i.e., the combination of C_3_ metabolism in non-succulent leaves and the Crassulacean Acid Metabolism (CAM) pathway in succulent stems, could efficiently maximize biomass accumulation^7,16,17^. Specifically, C_3_ promotes growth under favorable conditions, while non-succulent C_3_ leaves die quickly and CAM plays a critical role under drought stress, which could prevent damage from water limitation and ensure photosynthetic integrity^16^.

Due to the global energy crisis with the conventional fossil fuels and the associated environmental degradation^18–21^, *E. tirucalli* has received increasing attention in recent years, thanks to its fascinating petrochemical values and high tolerance to extreme habitats^4,5,22,23^. However, the utilization of such an important plant resource is severely hampered by the unavailability of genomic data. Therefore, a high-quality assembled genome of *E. tirucalli* is urgently required to uncover the genetic basis of both biodiesel production and stress resistance.

In this study, we performed a *de novo* chromosome-level genome assembly and annotation of *E. tirucalli* using PacBio HiFi sequencing and high-throughput chromosome conformation capture (Hi-C) technology. The assembled genome size of *E. tirucalli* was 745.62 Mb, with a contig N50 of 74.16 Mb. A total of 743.63 Mb (99.73%) of the assembled sequences were anchored to 10 chromosomes with a complete BUSCO score of 97.80%. The genome annotation identified 26,304 protein-coding genes. The high quality genome provides valuable genetic resources for further research on the genetic mechanisms underlying biofuel synthesis and adaptation to harsh conditions in *E. tirucalli*.

## Methods

### Sampling and sequencing

Fresh leaves of *E. tirucalli* were collected for whole genome sequencing from a healthy tree planted in the South China National Botanical Garden (accession number: IBSC0312991) (Fig. 1a). Additionally, tender leaves, mature leaves, young succulent stems, old stems, flowers, and roots were collected for transcriptome sequencing. All samples were immediately frozen in liquid nitrogen and stored at −80 °C.

Total genomic DNA was extracted using a cetyltrimethylammonium bromide (CTAB) method^24^. DNA quantity and quality were determined using a Qubit 4.0 fluorometer. Short read sequencing libraries with ~350 bp insert size were constructed and sequenced on the Illumina NovaSeq 6000 platform to generate 150 bp read pairs. For PacBio SMRT sequencing, a 15 kb DNA PacBio HiFi library was generated using the SMRTbell Express Template Preparation Kit 2.0. The library was then sequenced on the PacBio Sequel II platform, yielding 41.54 Gb of HiFi data with 53.96 × coverage. The Hi-C libraries were prepared by chromatin crosslinking, restricted enzyme (MboI) digestion, end filling and biotinylation tagging, DNA purification and shearing. All of the prepared DNA fragments were processed into paired-end sequencing libraries. Finally, a total of ~100 Gb of 150 bp paired-end Hi-C reads were obtained from the Illumina platform. For transcriptome sequencing, the RNA-seq libraries were constructed and then sequenced on the Illumina NovaSeq 6000 platform with 150 bp paired-end reads, generating about 18.8 Gb of RNA-seq reads.

### Genome survey for genome size estimation

A total of 35 Gb of clean data from the Illumina platform was used for the genome survey. Genome size, heterozygosity and repeat content were estimated from the *k*-mer frequency distribution using GCE^25^. The 17-mer analyses yielded an estimated genome size of 789.98 Mb, with heterozygosity of 0.80% and repeat content of 74.67% (Fig. 1b).

### *De novo* genome assembly

The PacBio HiFi reads were initially assembled into contigs using hifiasm v0.16.1-r375^26^ with default parameters. This analysis resulted in an assembly of 745.62 Mb and a N50 length of 74.16 Mb (Table 1). The size of the assembled genome is slightly smaller than our estimates (~760 Mb) by flow cytometry using *Oryza sativa* ssp. *japonica* (1 C = 0.43–0.45 pg) as a reference standard (Fig. S1). The completeness of the genome assembly was assessed using Benchmarking Universal Single-Copy Orthologues (BUSCO v5.4.3)^27^ with the embryophyta_odb10, which generated 97.8% of the Plantae BUSCO genes (Table 1). The accuracy of the draft assembly was further evaluated by mapping short reads to the genome assembly using the BWA-MEM v0.7.17-r1188^28^, which yielded a mapping rate and genome coverage of 99.88% and 99.75%, respectively (Table 1). For pseudochromosome construction, Hi-C reads were aligned to the contig-level assembly using Juicer v1.6^29^ with default parameters. We then used the 3D-DNA v201008 pipeline^30^ to correct mis-joins, anchor, order, and orient the assembled contigs. Manual inspections and adjustments of the draft assembly were performed using Juicebox v1.11.08^31^. Finally, approximately 743.63 Mb of scaffold was anchored to 10 pseudochromosomes, accounting for 99.73% of the assembled genome size (Fig. 1c,d). To evaluate the continuity of the genome assembly, the long terminal repeat retrotransposons assembly index (LAI), a reference-free genome metric for assessing the assembly of repeat sequences^32^ was calculated. The LAI value of the genome assembly was 19.05, which was close to the quality standard of a gold genome (LAI > 20) proposed by Ou *et al*.^32^. Collectively, these results validated the high completeness and reliability of our *E. tirucalli* genome assembly.

**Table 1:** Statistics of the *Euphorbia tirucalli* genome assembly and annotation.

Genome assembly	
Total length of assembly (Mb)	745.62
Number of contigs	123
Contig N50 (Mb)	74.16
Pseudochromosome number	10
Scaffold N50 (Mb)	75.09
Number of gaps	43
Anchor rate (%)	99.73
Mapped Illumina reads (%)	99.88
BUSCO (%)	97.80
GC content (%)	37.20
LAI	19.05
QV	67.58
K-mer completeness	87.71
**Genome annotation**	
Total length of repeats (Mb)	569.43
Repeats percentage of assembly (%)	76.37
Number of protein-coding genes	28,840
Average gene length (bp)	4,191.84
Average intron length (bp)	684.30
Average exon length (bp)	290.86
**Functional annotation**	
SwissProt	21,030 (72.92%)
Nr	26,252 (91.03%)
InterPro	23,340 (80.93%)
Pfam	21,186 (73.46%)
eggNOG	23,838 (82.66%)
Total	26,304 (91.21%)

### Repeat annotation

Tandem repeats were identified by *ab initio* prediction using TRF v4.09^33^. We used both *de novo* and homology predictions to annotate transposable elements (TEs) throughout the genome. For the homology-based strategy, RepeatMasker^34^ and RepeatProteinMask^34^ were used to extract the known repeat sequences. For *de novo* prediction, long terminal repeat (LTR) elements were first identified with LTR_FINDER v1.0.6^35^, LTRharvest v1.5.10^36^ and LTR_retriever^37^. Then, MITE-hunter^38^ and RepeatModeler^39^ were used for *de novo* repeat discovery. The MITE and consensus repeat libraries generated by RepeatModeler were combined and subjected to RepeatMasker for final repeat identification. Overall, 569.43 Mb (76.37%) of the assembly was masked as repeats. Of these, 75.70% were TEs, including long terminal repeat retrotransposons (LTR-RTs) (61.11%), non-LTR-RTs (1.10%), and DNA transposons (6.56%) (Fig. 1c; Table 2).

**Table 2:** Statistics of repeat sequences in the *Euphorbia tirucalli* genome.

Class	Length (bp)	Percentage in genome (%)
Class I: Retrotransposon	463,901,118	62.21
LTR Retrotransposon	455,692,982	61.11
Copia	208,117,659	27.91
Gypsy	242,670,614	32.55
Others	4,904,709	0.65
Non- LTR Retrotransposon	8,208,136	1.10
LINE	8,208,136	1.10
Class II: DNA Transposon	48,899,750	6.56
CMC-EnSpm	1,271,052	0.17
MITE	33,414,101	4.48
MULE-MuDR	6,018,597	0.81
PIF-Harbinger	1,683,188	0.23
TcMar-Pogo	159,571	0.02
hAT-Ac	1,721,574	0.23
Helitron	2,572,374	0.35
Unclassified	51,602,006	6.92
Total TE	564,402,874	75.70
Tandem repeat	5,027,446	0.67
Total repetitive sequences	569,430,320	76.37

### Gene prediction and functional annotation

Gene prediction was performed using a combined strategy of homology-based, *ab initio*, and RNA-Seq-assisted predictions. In detail, Trinity v2.15.0^40^ was used to *de novo* assemble the transcriptome for RNA-Seq-assisted prediction. Hisat2 v2.2.1^41^ was utilized to map RNA-seq reads to the genome, and Samtools v1.9^42^ was used to generate BAM file. Then Trinity and StringTie^43^ were utilized to assemble the genome-guided transcriptomes. The *de novo* and genome-guided transcriptome assemblies were merged, generating transcript evidence using PASA^44^. SNAP^45^, GeneID^46^, GlimmerHMM^47^, GeneMark-ET^48^ and AUGUSTUS^49^ were used for *ab initio* prediction with RNA-Seq-based predicted genes as training data. Homologies from five Euphorbiaceae species (*E. lathyris*, *E. peplus*, *Ricinus communis*, *Hevea brasiliensis*, and *Manihot esculenta*) and *Arabidopsis thaliana* were used as protein evidence for predicted gene sets using GeMoMa^50^. Finally, the results from the above three approaches were integrated using EVidenceModeler^51^ and further polished using PASA^44^. A total of 28,840 protein-coding genes were successfully predicted for *E. tirucalli*, with the average gene, intron and exons lengths of 4191.84 bp, 684.30 bp and 290.86 bp, respectively (Table 1).

For the functional annotation of protein-coding genes, a BLASTP (E-value ≤ 1e^−5^) search with the best match parameters was performed against publicly available protein databases of SwissProt and NR. Motifs and domains were annotated using InterProScan^52^ by searching against InterPro and Pfam. Gene ontology (GO) terms of the annotated peptide sequences were obtained using eggNOG-mapper v2.1.5^53^. Finally, 26,304 protein-coding genes were functionally annotated, representing 91.21% of the total predicted genes (Table 1).

## Data Records

The raw sequence data (Illumina, PacBio, Hi-C) have been uploaded to NCBI Sequence Read Archive (SRA) database with accession number SRR27885842^54^, SRR27885834^55^, and SRR27885835^56^, respectively, under the BioProject accession number PRJNA1070402. The RNA-seq data for different tissues are also available under PRJNA1070402 with accession numbers SRR27885836^57^, SRR27885837^58^, SRR27885838^59^, SRR27885839^60^, SRR27885840^61^, SRR27885841^62^. The final chromosome assembly has been deposited in NCBI GenBank with accession number JAZDXJ000000000^63^. The genome annotation file has been deposited in the Figshare database^64^.

## Technical Validation

We assessed the quality of the genome assembly in the following aspects: (1) Genome completeness was assessed by BUSCO v5.4.3^4^. The results indicated that 97.8% complete BUSCO genes were identified in the final assembly, of which 95.6% were single-copy, 2.2% were duplicated, and 0.4% were fragmented. (2) Mapping short reads to the genome assembly, which revealed a mapping rate and genome coverage of 99.88% and 99.75%, respectively. (3) The LTR Assembly Index (LAI) of the genome assembly is 19.05, which is close to the quality of a gold genome according to the classification system^32^. (4) Quality value (QV) and k-mer completeness were estimated using Merqury v1.3^65^, resulting in a QV of 67.58 and completeness of 87.71%. These results indicate that the *E. tirucalli* genome assembly is of high quality.

### Supplementary information

Fig. S1. Fluorescence histograms of flow cytometry for *Euphorbia tirucalli*.

## Data Availability

All software and pipelines used in this study were implemented according to the manuals and protocols provided by the software developers. Versions of the software have been described in Methods. No custom code was used in this study.
