# Supplementary material for: Chromosome-level genome assembly of Euphorbia tirucalli (Euphorbiaceae), a highly stress-tolerant oil plant
Source: Sci Data. 2024 Jun 21;11:658. doi: 10.1038/s41597-024-03503-w (PMC11192743; doi:10.1038/s41597-024-03503-w)
Supplement: Supplementary file 1 — Fig. S1. Fluorescence histograms of flow cytometry for Euphorbia tirucalli. [file 41597_2024_3503_MOESM1_ESM.pdf]

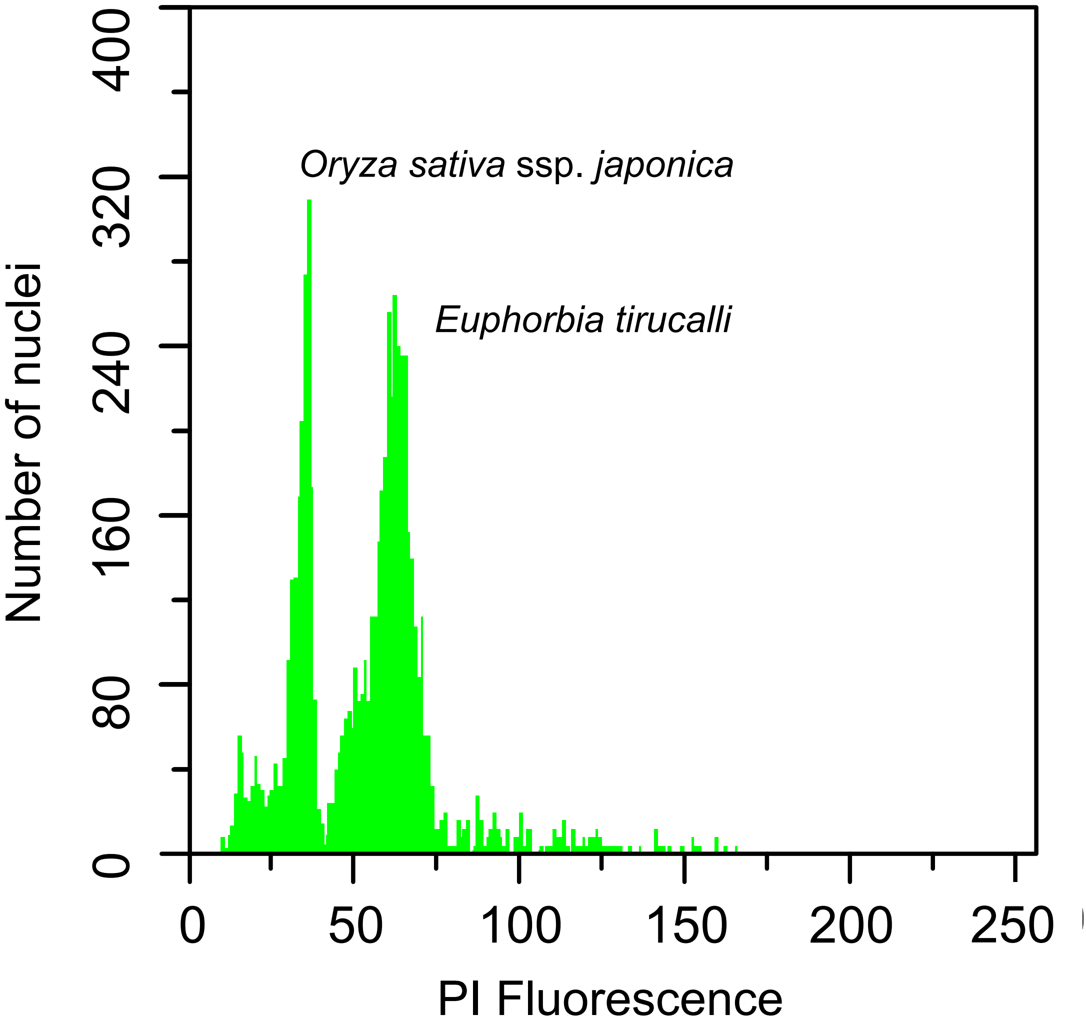

Fig. S1. Fluorescence histograms of flow cytometry for *Euphorbia tirucalli*. *Oryza sativa* ssp. *japonica* was used as a reference standard.
